# Biological reconstruction in the growing child after tumour resection: long-term outcomes of vascularized fibular epiphyseal transfer

**DOI:** 10.1007/s00264-026-07005-9

**Published:** 2026-08-24

**Authors:** Adyb - Adrian Khal, Lucas Lo Cunsolo, Virginie Rampal - Rocher, Jean - Luc Jouve, Ovidiu - Gogu Cacuci, Sebastien Pesenti

**Affiliations:** 1 Faculty of Medicine, University of Oradea, Oradea, Romania; 2 Department of Orthopaedics, Pelican Medicover Hospital, Oradea, Romania; 3 Department of Paediatric Orthopaedics, AP-HM Timone Enfants, Marseille, France; 4 Laboratoire Motricité Humaine Expertise Sport Santé (LAMHESS, EA-6312), UFR STAPS, Nice, France; 5 Department of Orthopedics, Lenval University Children’s Hospital, Nice, France

**Keywords:** Vascularized fibula epiphyseal transfer, Bone sarcoma, Pediatrics, Orthopedics

## Abstract

**Purpose:**

Paediatric limb-salvage surgery after tumour resection is challenging because reconstruction must account for small skeletal dimensions, immature bone, and residual growth. This study aimed to evaluate the long-term clinical, radiological, functional, and oncological outcomes of vascularized fibular epiphyseal transfer (VFET) after paediatric oncological resection.

**Methods:**

We retrospectively reviewed nine children who underwent VFET between 1995 and 2025 at two institutions. Surgical techniques, bone union, graft hypertrophy, neo-joint remodelling, growth-related changes, complications, functional outcomes, and oncological status were analysed.

**Results:**

The mean age at surgery was 6.8 years (range, 2–14), and the mean follow-up was 105 months (range, 7–330). Reconstruction sites included the proximal humerus, proximal radius, proximal femur, and distal tibia. Mean resection length was 12.25 cm. Bone union was achieved in seven patients after a mean of 22 months. Graft hypertrophy was observed in all patients at one year, with a mean increase of 34%. Neo-joint remodelling occurred in three patients. Two patients developed proximal humeral graft overgrowth with instability and dislocation. Mechanical complications occurred in eight patients, mainly non-union and graft fracture. One-year complication-free survival was 33 ± 16%. Functional outcomes were better in the upper limb than in the lower limb. Three-year overall survival was 71 ± 18%.

**Conclusions:**

VFET is a demanding biological reconstruction option in growing children after tumour resection. Although graft hypertrophy and selected joint remodelling may occur, growth behaviour is unpredictable and mechanical complications are frequent.

## Introduction

Primary malignant bone and soft tissue tumours are rare lesions, but a substantial proportion of bone sarcomas occur in children [1, 2]. In recent decades, advances in diagnostic imaging, chemotherapy, radiotherapy, surgical techniques, and reconstructive options have made limb salvage the preferred treatment whenever oncologically feasible [1, 3]. However, in children, limb-salvage surgery remains particularly challenging because reconstruction must be adapted to small skeletal dimensions, immature bone, residual growth, and long-term functional expectations [4].

These difficulties are even greater when tumour resection involves the metaphyseal or epiphyseal region. In such cases, reconstruction must not only restore bone continuity, but also address joint function, limb growth, and the risk of long-term deformity or impairment. Several reconstructive options may be considered after paediatric oncological resection, but none can be considered ideal in all situations. Endoprosthetic reconstruction, osteoarticular grafts, intercalary grafts, or vascularized fibular diaphyseal grafts may each be limited by patient age, bone size, fixation difficulties, expected growth, mechanical complications, or long-term durability.

The fibula is considered an expendable bone and has been widely used as a non-vascularized or vascularized graft for skeletal reconstruction. Taylor et al. [5] first described the free vascularized bone graft, and Weiland et al. [6] subsequently reported its use in malignant or aggressive bone tumours. The concept of transferring a growing physis is much older: Helferich [7] reported experimental epiphyseal transplantation in 1899, and Staub [8] later described a clinical free epiphyseal transfer. In the late 1980 s, Tsai et al. [9] reported one of the first clinical series of vascularized fibular epiphyseal transfer (VFET).

VFET is a rare biological reconstruction technique that aims to restore skeletal continuity while transferring a living growth plate with potential for hypertrophy, longitudinal growth, and neo-joint remodelling. Since its first clinical descriptions, several authors have reported its use after paediatric tumour resection, including complex reconstructions around the hip, shoulder, wrist, elbow, and ankle [4, 10–14]. However, despite its theoretical advantages, VFET remains controversial because of its technical complexity, unpredictable growth behaviour, and high rate of complications. Published results remain limited, and no consensus has been reached regarding the ideal indications for this demanding reconstruction [10–14].

Long-term evaluation is essential because the success of VFET cannot be assessed only by early graft survival or bone union. Its real value depends on graft hypertrophy, growth behaviour, neo-joint remodelling, mechanical durability, functional outcome, and the burden of secondary procedures until skeletal maturity and beyond. Better understanding of these outcomes may help surgeons select appropriate indications, counsel families more accurately, and balance the potential benefits of biological reconstruction against the risk of repeated surgery.

Therefore, the aim of the present study was to evaluate the long-term clinical, radiological, functional, and oncological outcomes of children who underwent VFET after oncological resection, with particular attention to bone union, graft hypertrophy, neo-joint remodelling, growth-related changes, mechanical complications, and functional outcome.

## Materials and methods

We retrospectively analyzed all medical records of paediatric patients who underwent oncological resections and reconstruction with VFET between January 1995 and December 2025 at two centers. We identified and included in the study nine patients with at least two years of follow-up, except one patient who died of disease after seven months.

For each patient, medical and surgical (both orthopedic and plastic surgery) reports were reviewed to identify reconstructive indications, technical details, and eventual oncological management. The surgical technique followed that of Hariri et al. [15], wherein a vascularized fibular segment was harvested by one or two pedicles and that of Kanazawa et al. [16], wherein the knee was stabilized by reattaching the biceps tendon to the tibia with a screw or with osteo-sutures. The graft was harvested contralaterally in the case of upper limb reconstruction and ipsilaterally in the case of lower limb reconstruction.

The use of radiotherapy and chemotherapy was decided at the discretion of a multidisciplinary team (orthopedic surgeon, radiotherapist, and medical oncologist). One patient underwent adjuvant radiation therapy (54 Gy) after resection of a synovial sarcoma.

To evaluate surgical margins, resected specimens were categorized as radical, wide, marginal, and intralesional according to the Enneking criteria [17].

Patients were evaluated every three months during the first two years, every six months up to five years after surgery, and once a year until ten years after surgery. All complications were recorded. Clinical examination included stability assessments of the above and below joints, the joints’ range of motion, and walking.

Radiographic evaluation included full weight-bearing long-leg radiographs and focal radiographs. Bone union was considered when a bone bridge between the transplant and recipient bone was present and the osteotomy line had disappeared. Graft hypertrophy was measured according to the De Boer–El-Gammal formula [18, 19] at one year on the anteroposterior radiographic view, and was considered acquired when > 20%. Articular remodeling was assessed as present or absent according to the congruence of the neo-joint. Image findings were evaluated independently and cross-checked by two observers (K.A.A. and L.L.C.) blinded to all other patient information. 

The Musculoskeletal Tumor Society Score (MSTS) [20] was used to evaluate the functional outcome at the last follow-up.

Data are presented as means (range), total frequencies, and percentages. Kappa values were calculated to evaluate the intra-observer (one-month interval) and inter-observer errors. Intra-observer and inter-observer agreements were considered when the kappa value exceeded 0.60. All radiographic values presented relate to the last assessment of an observer. For oncological outcomes, the overall survival (OS) rate was calculated as the time from diagnosis to death from any cause, and complication-free survival (C-FS) was calculated as the time from the surgery to distant complication or death from any cause, using Kaplan–Meier analysis. For univariate analysis of oncological outcomes, differences between curves were analyzed using the log-rank test. The chi-squared test was used to compare categorical variables. Statistical analyses were performed using SPSS Statistics version 26 (IBM Corp., Armonk, NY, USA). Statistical significance was set at p < 0.05.

## Results

Nine children underwent VFET after oncological resection during the study period. Reconstruction sites included the proximal humerus in five patients, proximal femur in two patients, distal tibia in one patient, and proximal radius in one patient. There were three boys and six girls, with a mean age at surgery of 6.8 years (range, 2–14 years). The mean follow-up was 105 months (range, 7–330 months) [Table 1].

**Table 1:** Demographic data for patients included in the study

Patient #	Age and sex at surgery	Lesion	Site	Resection length (cm)	Fibula graft length (cm)	Bone union (months)	Pseudoarthrosis (months)	Fracture (months)	LLD (cm)	Other complications (x, months)	Follow-up time (months)	MSTS score	Patients’ status
1	8, F	OstS	Proximal humerus	18	20	No	Yes, 7	-	-	Metastasis, 3	7	21	DOD
2	2, M	OstS	Distal tibia	9.5	11	17	Yes, 14	Yes, 24	Yes, 2.5	-	119	26	NED
3	6, M	OstS	Proximal femur	11	15	9	-	-	-	Metastasis, 28	38	10	DOD
4	14, F	SS	Proximal radius	6	7	18	Yes, 3	-	-	-	24	23	NED
5	10, M	OstS	Proximal humerus	12	13	No	Yes, 14	-	-	-	24	21	NED
6	9, F	EwS	Proximal humerus	18	19	15	Yes, 13	Yes, 12	-	Aseptic wound dehiscence	25	23	NED
7	4, F	EwS	Proximal femur	10	11	4	-	Yes, 3, 98	-	-	330	12	NED
8	3, M	EwS	Proximal humerus	15	16	9	Yes, 5	Yes, 188	-	-	208	18	NED
9	6, F	OstS	Proximal humerus	8	9	82	Yes, 10	-	Yes, 4	Deep infection	174	19	NED

LLD = Limb length discrepancy, MSTS = Musculoskeletal Tumor Society Score, M = male, F = female, OstS = Osteosarcoma, SS = Synovial Sarcoma, EwS = Ewing Sarcoma, DOD = died of disease, NED = no evidence of disease.

### Patient and reconstruction characteristics

**Fig. 1:**
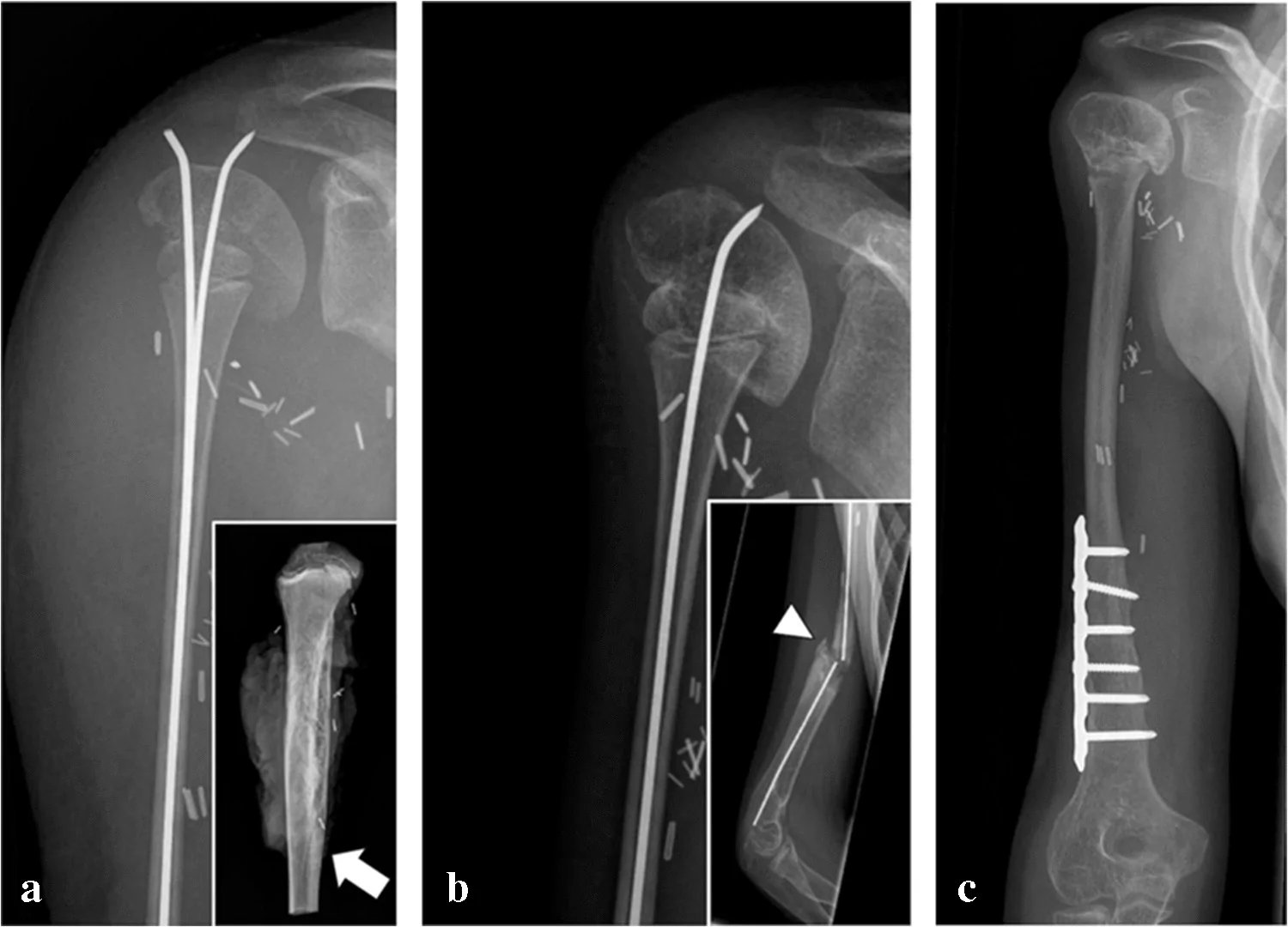
Radiographs of the proximal humerus of a nine-year-old girl show articular remodelling after VFET. (**a**) Immediate postoperative anteroposterior radiograph after proximal humeral resection, with preservation of a thin osteocartilaginous layer of the proximal humeral epiphysis; one K-wire was removed after migration at three months. (**b**) One year later, fracture of a K-wire occurred at the host–graft junction. (c) The fracture was treated with plate fixation without compromising articular remodelling; progressive graft hypertrophy is visible six months later

Tumour diagnoses included osteosarcoma in five patients, Ewing sarcoma in three patients, and synovial sarcoma in one patient. Surgical reconstruction consisted of isolated VFET in all patients; however, in one patient, resection permitted preservation of a thin osteocartilaginous layer of the epiphysis (Fig. 1). The mean resection length was 12.3 cm (range, 6–18 cm), and the mean graft length was 13.4 cm (range, 7–20 cm).

Vascular anastomosis was performed between one major recipient artery and the peroneal artery in six patients, the anterior tibial artery in two patients, and both the peroneal and anterior tibial arteries in one patient. All patients received subcutaneous heparin at 50 IU/kg twice daily and oral aspirin 75 mg for one month postoperatively. No complete or transient peroneal nerve palsy related to fibular harvesting was observed. Surgical margins were negative in all patients.

### Bone union, graft hypertrophy, and neo-joint remodelling

Bone union was achieved in seven patients (78%) after a mean of 22 months (range, 4–82 months). The intra-observer kappa value for bone union was 0.60, and the inter-observer kappa value was 0.72. In two patients, bone union occurred without secondary surgery, after four and nine months, respectively.

**Fig. 2:**
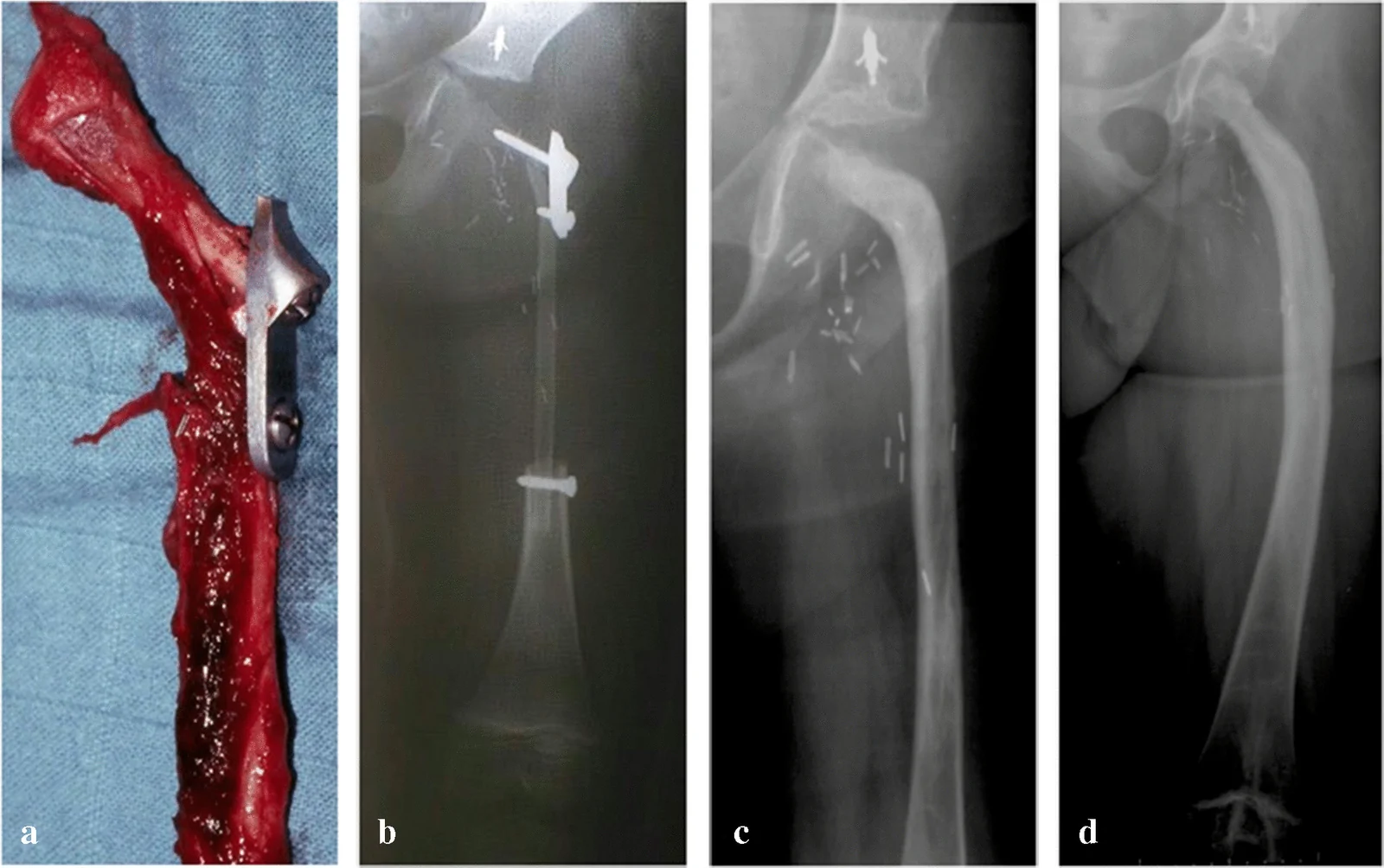
Radiographs of the proximal femur of a four-year-old girl show progressive anatomical and mechanical remodelling after VFET. (**a**) Intraoperative view of the transferred graft. (**b**) Immediate postoperative anteroposterior radiograph after medial closing-wedge osteotomy and fixation with a custom-made plate to reproduce the femoral neck; the periosteum was preserved and medialised. (**c**) Radiograph after plate removal following metaphyseal union at two years. (**d**) At eight years of follow-up, remodelling of the reconstructed proximal femur is visible

**Fig. 3:**
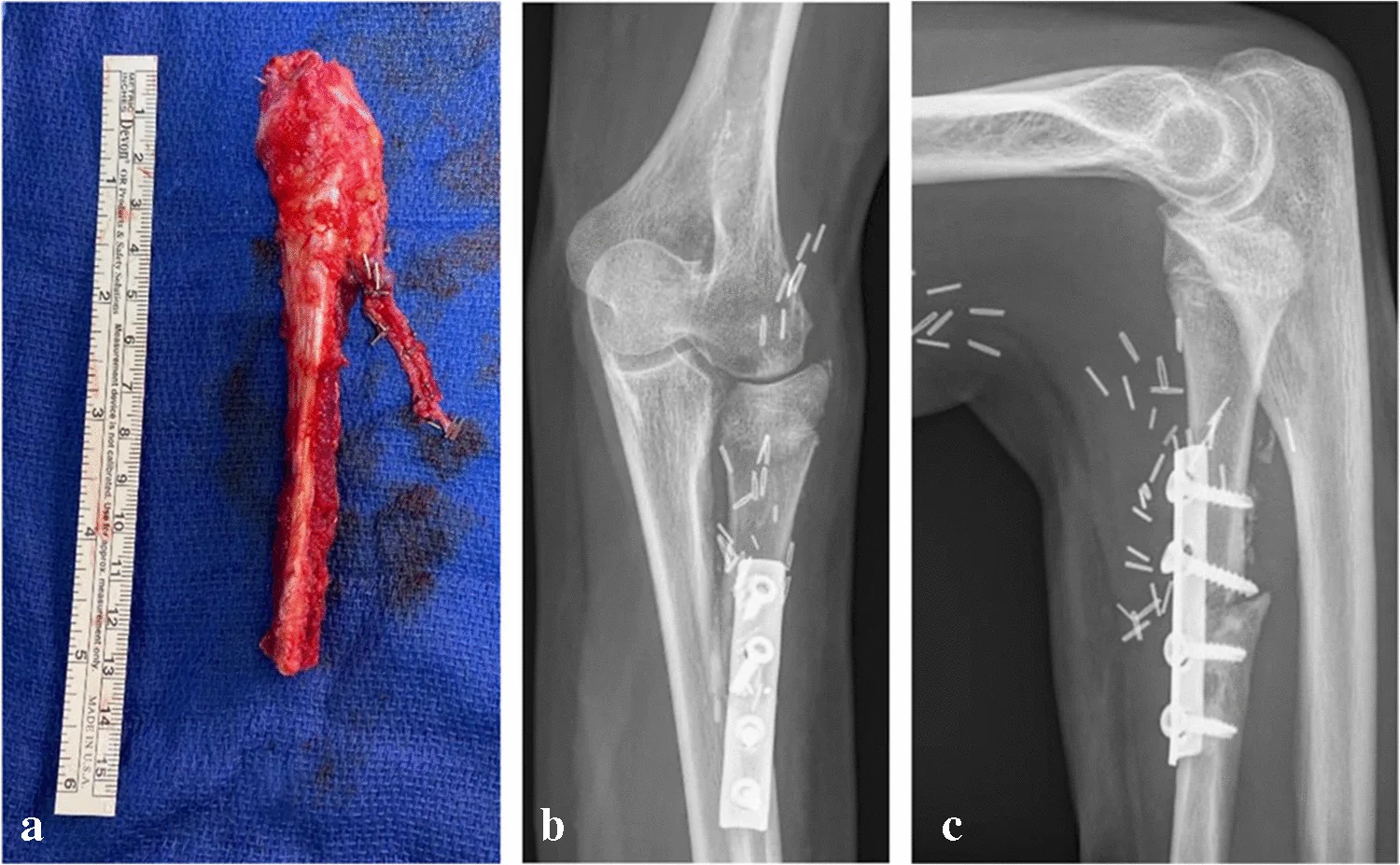
Radiographs of the proximal radius of a 14-year-old girl show neo-joint remodelling after VFET for synovial sarcoma resection. (**a**) Harvested VFET used for proximal radial reconstruction. (**b**, **c**) Anteroposterior and lateral elbow radiographs at 14 months show joint remodelling despite persistent visibility of the osteotomy line

Graft hypertrophy greater than 20% was observed in all patients at one year. The mean graft hypertrophy was 34% (range, 25–65%). Neo-joint remodelling was observed in three patients (33%). The intra-observer kappa value for remodelling was 1, and the inter-observer kappa value was 0.75. Remodelling was observed after reconstruction of the proximal humerus (Fig. 1), proximal femur (Fig. 2), and proximal radius (Fig. 3).

### Growth-related changes and mechanical complications

Growth behaviour was variable and could not be quantified globally because standardised serial radiographs were not available for all patients. Growth arrest occurred in three patients. Two patients had residual limb-length discrepancies of 2.5 cm and 4 cm after additional procedures. Two patients developed proximal humeral graft overgrowth with instability and dislocation; both were subsequently treated with arthrodesis. In one patient with proximal femoral reconstruction, the transferred epiphysis became unstable and migrated within the iliac bone.

Mechanical complications occurred in eight patients (89%). The one-year complication-free survival rate was 33 ± 16%. Six consolidation defects required revision surgery after a mean of nine months (range, 3–14 months), with favourable subsequent progression. Isolated or multiple graft fractures occurred in four patients (44%) after a mean of 65 months (range, 3–188 months). Three fractures occurred late, at two years, 8.2 years, and 15.6 years of follow-up, despite radiographic bone healing.

### Functional and oncological outcomes

The mean MSTS score at last follow-up was 19 points (range, 10–26). Functional outcomes were better after upper-limb reconstruction than after lower-limb reconstruction, with mean MSTS scores of 20.8 points (range, 18–23) and 16 points (range, 10–26), respectively. Patients with neo-joint remodelling had variable functional outcomes, including an MSTS score of 13 after proximal femoral reconstruction and 23 after proximal humeral reconstruction.

No patient had metastasis at diagnosis, and no local recurrence was observed. Two patients developed pulmonary metastases at three and 29 months postoperatively and died of disease at seven and 38 months of follow-up, respectively. The three-year overall survival rate was 71 ± 18%.

## Discussion

Paediatric limb-salvage surgery remains challenging because reconstruction must be adapted to smaller skeletal dimensions, immature bone, and residual skeletal growth [4]. VFET has biological and mechanical advantages because it may provide a living graft capable of hypertrophy, remodelling, and potential longitudinal growth, but these advantages must be balanced against a high rate of complications [10, 21]. Few studies have described this complex reconstructive technique in children, and the reported results remain variable [4, 10–12, 21–28]. The present retrospective study describes the long-term clinical, radiological, and functional outcomes of VFET after paediatric oncological resection. In our series, graft hypertrophy was observed in all patients at one year, and neo-joint remodelling was observed in selected cases. However, growth behaviour was unpredictable, and mechanical complications were frequent. Consistent with previous studies, these patients often required additional surgery, although acceptable limb function could still be achieved in selected anatomical locations [10, 21, 23, 29].

The main published studies related to VFET are summarised in Table 2. Most available reports are small case series or reviews, often focused on a single anatomical site, which reflects the rarity of the technique and explains the absence of clear consensus regarding indications and expected outcomes.

**Table 2:** Summary of the main published clinical series on vascularized fibular epiphyseal transfer in paediatric oncological reconstruction

Study	Patients (n =)	Reconstruction site (n =)	Mean Age	Mean Follow-up	Union/hypertrophy/growth	Complications	Functional outcome
Innocenti et al. [11]	12	Proximal humerus (9) Distal radius (3)	5,6	16 months	All grafts survived and united within 3 months; ATA-based graft growth 0.75–1.33 cm/year; premature physeal fusion in 2 peroneal artery grafts	Premature physeal fusion n = 2; subacromial displacement n = 2	Excellent distal radius results when ulna spared; humeral function acceptable for daily activities but limited shoulder motion
Innocenti et al. [23]	8	Distal radius (8)	7.3	13.2 years	All grafts survived and united; expected growth 0.7–1.4 cm/year; progressive joint remodelling; growth arrest n = 1	No major recipient-site complications; peroneal nerve palsy n = 3, persistent n = 1	Excellent in all but one patient; long-term pain-free joint without degenerative changes
Shammas et al. [12]	4	Proximal humerus (4)	7.5	88.9 months	Union in all patients; mean growth 0.54 ± 0.18 cm/year; mean hypertrophy 47.7%	Graft fracture n = 4/4; peroneal nerve symptoms n = 4/4; persistent deficit n = 1	Preserved elbow and hand function; no active shoulder motion
Stevenson et al. [21]	11	Proximal humerus (11)	5	5.2 years	Hypertrophy/growth in 9 grafts; mean hypertrophy 65%; mean growth 4.6 mm/year	Fracture n = 7; transient nerve palsy n = 4; AVN n = 2; epiphyseal slip n = 1; tibial physeal arrest n = 1	Mean MSTS 77%; mean TESS 84%
Bachy et al. [10]	7	Proximal femur (3), Proximal humerus (3), Distal radius (1)	5.7	11 years	Integration in all cases; recovered growth in 4/7	Infection n = 1; consolidation defects n = 2; fractures n = 8; malalignment n = 1; transient peroneal palsy n = 2	Satisfactory joint function in 85%; lower-limb function more variable
Present study	9	Proximal humerus (5), Proximal femur (2), Distal tibia (1), Proximal radius (1)	6,8 years	105 months	Union 7/9; hypertrophy in all at 1 year; mean hypertrophy 34%; remodelling 3/9; growth arrest 3/9; overgrowth 2/9	Mechanical complications 8/9; non-union 6/9; fracture 4/9; LLD 2/9; instability/dislocation 2/9; infection 1/9	Mean MSTS 19; better upper-limb than lower-limb outcome, 20.8 vs 16

ATA = anterior tibial artery; AVN = avascular necrosis; LLD = limb-length discrepancy; MSTS = Musculoskeletal Tumor Society score; TESS = Toronto Extremity Salvage Score; VFET = vascularized fibular epiphyseal transfer.

In our practice, the choice of reconstruction after paediatric tumour resection depends on patient age, tumour site, expected residual growth, defect size, joint involvement, soft-tissue sacrifice, adjuvant treatment, and expected survival. In older children and adolescents, reconstructive options may include modular or expandable endoprostheses, osteoarticular or intercalary allografts, vascularized fibular diaphyseal grafts, or combined biological reconstructions. However, in very young children, these options may be limited by small bone size, fixation difficulties, implant longevity, risk of mechanical failure, and inability to preserve growth potential. VFET was therefore considered in highly selected cases in which reconstruction required a living biological graft with potential for hypertrophy, growth, and articular remodelling.

Biological reconstructions have several potential advantages in growing children. They use living autologous tissue, may adapt to mechanical loading, may hypertrophy over time, and may provide durable reconstruction in long-term survivors. In the specific setting of VFET, transfer of a vascularized physis may also provide growth potential and, in selected cases, contribute to remodelling of a neo-joint. However, these reconstructions are technically demanding and expose patients to prolonged healing, delayed union, non-union, graft fracture, instability, deformity, and secondary procedures. These disadvantages were evident in our series, in which mechanical complications occurred in eight of nine patients.

Like all large skeletal reconstructions, VFET is associated with a high rate of complications. Our overall mechanical complication rate was 89%, which is comparable with previous studies reporting complication rates of 40–85% [22, 29]. Fracture and non-union are among the most frequent complications after VFET [21, 29]. In the upper limb, the reported fracture rate varies between 20 and 41%, whereas in the lower limb it is approximately 35% [13, 30]. These values are similar to those reported after intercalary reconstruction with vascularized fibular diaphyseal grafts [13, 30]. Fractures may occur regardless of fixation method, as they have been reported with external fixation, intramedullary nailing, locking plates, or screws alone [14]. The tubular shape of the fibula, the mismatch between graft and recipient bone diameter, fixation constraints in small children, and the prolonged time required for adaptation and hypertrophy may contribute to this risk [22]. Importantly, late fractures may also occur despite apparent union and graft integration [10]. In our series, three late fractures after minor trauma occurred at two years, 8.2 years, and 15.6 years of follow-up despite radiographic bone healing.

Vascularized grafts have variable consolidation rates, with reported union occurring after a mean of 2.4–7.7 months [23, 25, 31]. In children, graft non-union has been considered less frequent than in adults, but it remains a major issue after VFET. In our series, 67% of patients required surgical revision for consolidation defects after a mean of 7.6 months. Several factors may explain this high rate of non-union, including graft length, recipient site, fixation method, vascular pedicle configuration, adjuvant radiotherapy, and the mechanical environment of the reconstruction. Combined allograft or double-barrel graft techniques have been proposed to improve mechanical stability and reduce the risk of secondary procedures [32, 33].

Bone formation and resorption vary according to Wolff’s law [34]. When the fibula is transferred to another anatomical site, the graft and transferred epiphysis must adapt to new mechanical, axial, and joint constraints [13, 14, 21]. Bone and joint remodelling might be expected to be greater in the lower limb because of weight-bearing, but the difference in calibre between the fibula and recipient bone, particularly in the femur, may make uniform transformation difficult [13, 14]. In our series, remodelling was observed in selected cases and could be favoured by surgical adaptation of the graft, such as osteotomy and plate fixation to reproduce proximal femoral anatomy. However, radiological remodelling did not necessarily guarantee better function [4, 10]. In the upper limb, especially around the distal radius or proximal humerus, remodelling may be more favourable and may be associated with better function of the neo-joint [23, 35]. This may be related to anatomical differences between the deep concavity of the acetabulum and the flatter glenoid, as well as to the greater tolerance for motion compensation around the shoulder [14].

Previous reports support the concept that VFET may be useful in skeletally immature patients when residual growth is expected [12]. The reported annual longitudinal growth of the transferred fibular physis varies from 0.54 to 1.72 cm/year [12, 22, 23]. Several factors may influence graft growth, including patient age, recipient bone anatomy, vascular supply, local mechanical constraints, and chemotherapy [23]. It is also important to consider the growth of adjacent bones to avoid progressive deformity, particularly in two-bone segments such as the forearm [14]. In our study, global annual growth of the graft could not be quantified because standardized serial radiographs were not available for all patients. However, growth arrest occurred in three patients, residual limb-length discrepancy persisted in two patients, and two patients developed graft overgrowth in the proximal humerus with instability and dislocation. These findings suggest that transfer of a vascularized physis may preserve growth potential, but does not guarantee predictable or clinically useful growth.

At long-term follow-up, functional outcomes were variable. In the upper limb, our mean MSTS score was comparable to those reported by other authors (69% versus 77–80%) [21, 22, 31]. When the axillary nerve and deltoid muscle are preserved, other reconstructive options such as composite prosthesis, the Capanna technique, or modular endoprosthesis may provide better function. However, in very young children, fibular grafts may be more suitable for small bone dimensions and may offer a biological option when growth potential is important. In the lower limb, VFET may appear attractive because of the possibility of biological reconstruction, but the consequences of complications are greater because of weight-bearing demands, limb-length issues, alignment, and joint stability. Each mechanical complication may negatively affect the final MSTS score, emphasizing the difficulty of achieving an ideal reconstruction in this setting.

The oncological outcomes in this series should be interpreted cautiously. No local recurrence was observed, and the three-year overall survival was 71 ± 18%. However, this small cohort included different tumour diagnoses, anatomical sites, treatments, and follow-up durations. Oncological survival is primarily influenced by tumour biology, systemic treatment response, surgical margins, and metastatic progression, rather than by the reconstructive technique itself. Therefore, the oncological results are reported descriptively and should not be overinterpreted.

This study has several limitations. First, it is retrospective and includes a small number of patients, reflecting the rarity of this reconstruction but limiting statistical interpretation. Second, the cohort was heterogeneous, including different diagnoses, anatomical sites, ages, and reconstructive contexts. Third, there was no comparison group, preventing direct comparison with endoprosthetic reconstruction, allograft reconstruction, vascularized fibular diaphyseal grafting, or other biological techniques. Fourth, standardized long-term radiographs were not available for all patients, so longitudinal graft growth could not be reliably quantified. Fifth, the long inclusion period may have introduced variability in imaging quality, surgical technique, fixation methods, adjuvant treatment, rehabilitation, and follow-up protocols. Therefore, the results should be interpreted as descriptive long-term observations rather than evidence of superiority or reproducibility.

Despite these limitations, this study provides long-term information on a rare reconstructive technique in paediatric musculoskeletal oncology. Its main value lies in describing the long-term biological behaviour of VFET, including graft hypertrophy, selected neo-joint remodelling, growth disturbance, late mechanical complications, and functional variability. These findings may help surgeons counsel families more accurately and reserve VFET for carefully selected patients in whom the potential benefits of biological reconstruction justify the risk of repeated procedures.

## Conclusion

VFET is a demanding biological reconstruction technique that may provide graft hypertrophy and selected neo-joint remodelling in growing children after tumour resection. However, longitudinal growth was unpredictable, and mechanical complications and secondary procedures were frequent. VFET should therefore be considered a selective reconstructive option for carefully chosen paediatric patients rather than a uniformly reliable solution. Long-term clinical and radiological surveillance until skeletal maturity and beyond is essential.

## Data Availability

No datasets were generated or analysed during the current study.
